# Adherence barriers and facilitators for cervical screening amongst currently disadvantaged women in the greater Cape Town region of South Africa

**DOI:** 10.4102/phcfm.v5i1.492

**Published:** 2013-07-05

**Authors:** Chantelle De Abreu, Hannah Horsfall, Despina Learmonth

**Affiliations:** 1 Psychology Department, University of Cape Town, South Africa

## Abstract

**Background:**

In South Africa cervical cancer is the second most commonly occurring cancer amongst women, and black African women have the highest risk of developing this disease. Unfortunately, the majority of South African women do not adhere to recommended regular cervical screening.

**Objectives:**

The purpose of this research was to explore the perceptions, experiences and knowledge regarding cervical screening of disadvantaged women in two informal settlements in South African urban areas.

**Method:**

The Health Belief Model (HBM) provided a theoretical framework for this study. Four focus groups (*n* = 21) were conducted, using questions derived from the HBM, and thematic analysis was used to analyse the data. The ages of the women who participated ranged from 21 to 53 years.

**Results:**

The analysis revealed lack of *knowledge* about screening as a key structural barrier to treatment. Other structural barriers were: *time*, *age* at which free screening is available, and *health education*. The *psychosocial* barriers that were identified included: *fear* of the screening procedure and of the *stigmatisation* in attending screening. The presence of *physical symptoms*, the perception that screening provides *symptom relief*, HIV status, and the desire to know one's physical *health status* were identified as facilitators of cervical screening adherence.

**Conclusion:**

This knowledge has the potential to inform healthcare policy and services in South Africa. As globalisation persists and individuals continue to immigrate or seek refugee status in foreign countries, increased understanding and knowledge is required for successful acculturation and integration. Developed countries may therefore also benefit from research findings in developing countries.

## Abstrait

**Barrières d'adhérence et d'animateurs pour le dépistage du col utérin chez les femmes actuellement défavorisés de la région du Cap en Afrique du Sud plus**

**Origine:**

En Afrique du Sud, le cancer du col de l'utérus est le deuxième cancer le plus fréquent chez la femme, et les femme noires d'Afrique ont le plus grand risque de développer cette maladie. Malheureusement, la majorité des femmes sud-africaines n'adhèrent pas au dépistage régulier qui est recommandé.

**Objectifs:**

L'objectif de cette recherche était d'explorer les perceptions, les expériences et les connaissances du dépistage du col de l'utérus parmi les femmes actuellement défavorisées résidant dans deux colonies urbaines informelles en Afrique du Sud.

**Méthode:**

Le ‘Health Belief Model’ (HBM) a fourni un cadre théorique pour cette étude. Quatre groupes de discussion (*n* = 21), avec des femmes âgées entre 21 et 53 ans, ont été réalisées en utilisant des questions dérivant de l'HBM, et l'analyse thématique fut utilisée afin d'analyser les données.

**Résultats:**

L'analyse a révélé un manque de connaissances à propos du dépistage comme un obstacle majeur au traitement. D'autres obstacles structurels identifiés furent: le temps, l’âge auquel le dépistage gratuit est disponible et l’éducation sanitaire. Les obstacles psychosociaux qui furent identifiées sont: la peur du procéder de dépistage et la peur de la stigmatisation par rapport a la participation au dépistage. Finalement, la présence de symptômes physiques, la perception que le dépistage procure un soulagement des symptômes, le statut VIH, et le désir de connaître l’état de sa santé physique furent identifiés en tant que facilitateurs de l'adhésion de dépistage du col de l'utérus.

**Conclusion:**

Cette étude a mis en évidence de nombreux obstacles au dépistage du col de l'utérus, et a identifié plusieurs facteurs cruciaux afin d'améliorer la dévotion. Cette connaissance a le potentiel d'informer les mesures et les services de soins de santé en Afrique du Sud. Réduction de la stigmatisation et une éducation de santé culturellement spécifique promet d’être un moyen efficace afin d'augmenter la dévotion au dépistage du col de l'utérus des femmes actuellement défavorisées résidant dans les établissements informels urbains. Des changements des services de soins de santé afin d'abaisser l’âge auquel le dépistage gratuit est disponible sont également essentielles pour accroître la dévotion au dépistage parmi cette population défavorisée.

## Introduction

In South Africa cervical cancer is the second most commonly occurring cancer amongst women.^1^ Recent surveys indicate that 5743 South African women are diagnosed with cervical cancer annually. Despite readily available and effective treatment of early stages of the disease as well as the existence of reliable and accessible screening, where cervical cytology is used, 3027 (53%) of these women will die from this disease each year.1 In South Africa, the risk of disease differs for different ethnic groups.^2, 3^ Black African women have the greatest risk of developing cervical cancer, one in every 34 women developing this disease.^2^

The human papillomavirus (HPV) is recognised as being responsible for 62.8% of invasive cervical cancers.^1^ HPV is associated with a more rapid disease progression; it reduces disease development to as brief a period as 20 months.^4^ Alarmingly, approximately 21% of South African women are carrying this sexually transmitted infection.^1^

### Cancer Screening and Treatment

A three-year period of successful cervical cytology, using Papanicolaou (Pap-smear) screening, is estimated to reduce cervical cancer incidence by 60% – 90% in populations which have previously never undergone screening.^5^ This screening aims to reduce the mortality rate associated with the disease through the early detection and treatment of abnormalities in the cells lining the cervix.^6^

On receipt of abnormal Pap-smear results (e.g. results suggesting precancerous lesions) appropriate follow-up consists of repeat Pap-smears and, when necessary, repeat colposcopies together with biopsies of the abnormal areas.^7, 8, 9^ In contrast to this relatively simple treatment plan, far more drastic treatments are necessary when lesions are only detected once they have progressed to the stage of invasive cancer. In most cases, invasive cervical cancer treatment includes a radical hysterectomy and/or radiation therapy.^10^ It is widely accepted that the more advanced the cancer, the more costly the treatment and the poorer the prognosis. However, the differences in survival projections between the various stages of cervical cancer are of more concern.^10, 11^ With appropriate treatment, the five-year survival rate for women with invasive cervical cancer (e.g. stage I disease) is estimated to be between 80% and 90%. These rates decrease significantly, to between 50% and 60%, for women with stage II carcinomas and even more dramatically when moving to stage III and IV carcinomas, with survival rates of less than 30% and less than 15%, respectively.^10^

The above survival rates and treatment recommendations emphasise the importance of early detection and continued follow-up care in order to reduce avoidable morbidity and mortality in women suffering from cervical cancer.^12^ Unfortunately, the majority of South African women do not adhere to recommended cervical screening practises. A survey conducted by the World Health Organisation^1^ for the 2001–2002 period estimated that only 13.6% of South African women had received a Pap-smear in the previous three years.^1^

### Barriers and facilitators to cervical screening

#### Knowledge

Research has consistently shown misinformation and a lack of knowledge regarding cervical cancer and preventative screening to be major barriers to screening adherence.^11, 13, 14, 15, 16, 17^

An important aspect of misinformation is women's inability to distinguish between cervical screening and diagnostic tests. Research has repeatedly shown that women are frequently unaware of the purpose and significance of a Pap-smear. Many women do not understand the importance of the Pap-smear as a preventative measure and believe that Pap-smears detect existing cancer.^14, 15, 18^ Accompanying this belief is the notion that a Pap-smear is performed when a women is suffering from a reproductive health problem such as vaginal bleeding or discharge.^11, 15^ Consequently, women will often only undergo cervical screening once they are symptomatic.^19^ As a result, their cancer is only detected at a more advanced stage of the disease, which is associated with significantly lower survival rates and more costly and severe treatment plans.

Finally, research by Pillay^20^ highlights the importance of this barrier within the South African context. This study investigated the degree of awareness held by rural and urban disadvantaged South African women regarding cervical and breast cancer. The study found that 20% of the women had never heard of cervical cancer and that more than 50% were unaware of cervical screening tests.

#### Language

Research repeatedly sites language as a key barrier to cervical screening adherence.^6, 11, 14, 17, 19^ A common feature of populations with low screening adherence is that the women do not have access to healthcare providers who speak their first language. This results in inadequate communication of the purpose and importance of cervical screening procedures.^11, 14, 19^ Furthermore, media used to promote screening rarely caters for all language groups, and as a result, many women do not receive adequate information about cervical screening.^17^

#### Cultural beliefs and attitudes

Many studies cite culture as a crucial barrier to cervical screening.^11, 21, 22^ An important aspect of cultural beliefs and attitudes is the influence they exert on decision-making practices. Research shows that treatments which are markedly different from a woman's traditional practices are often not followed. Amongst various low-income cultural groups, especially African groups, a decision regarding medical treatment involves the input of the whole family. If a woman's family decides that a treatment does not fit in with her cultural practices, she may be barred from seeking further treatment.^11, 21, 22^ A woman's culture shapes the manner in which she understands and experiences cervical cancer and screening.^21^ It is therefore crucial to take the influence of culture into account when investigating cervical screening adherence within the culturally diverse and complex context of South Africa.

#### Health professional characteristics and influence

The vast majority of research into the barriers and facilitators of cervical screening adherence indicate that women's experiences with service providers greatly influence their adherence decisions.^11, 13, 14, 15, 16, 17, 18, 19, 23^ Women have reported that discourteous, insensitive healthcare providers are a major deterrent to undergoing screening.^13, 17, 19^ In addition, healthcare provider gender has a significant effect on adherence. Women frequently cite a lack of female providers as the primary reason for not attending cervical screening.^11, 13, 14, 18, 19^ Finally, a health professional's recommendation has been found to be a major facilitator of cervical screening adherence .^16^

#### Fear and anxiety

Fear and anxiety surrounding ideas of pain, embarrassment and a potential cancer diagnosis greatly contribute to the absence of screening adherence.^11, 13, 14, 16, 17, 18, 19, 24, 25^ Women frequently report a fear that the Pap-smear will be painful as well as a fear that they will experience pain or sustain internal injuries after the procedure.^11, 13, 14, 16, 18, 19, 24, 25^ Fear of embarrassment because of a loss of privacy when they have to expose their genitalia is another barrier repeatedly cited by women.^11, 13, 14, 16, 18^ Finally, a fear of receiving a cancer diagnosis has been identified as a key barrier to cervical screening. Many women have a fatalistic attitude to a cancer diagnosis and fear the potential treatment plans and associated costs and discomforts.^11, 13, 14, 17, 18, 19, 24^ The fears mentioned above place women in high states of anxiety, and as a result, many women do not at adhere to recommended screening practices.

#### Service accessibility and cost

Time forms a significant access barrier and includes factors such as a long waiting period at clinics, lengthy travelling times to clinics and inconvenient clinic operating hours.^11, 14, 16, 19^

Another access barrier which has consistently been shown to deter cervical screening adherence is the costs associated with screening.^11, 13, 16, 19, 24^ More specifically, research has shown that travel costs and procedural costs discourage women from undergoing screening. In contrast to this, the presence of health insurance and free cervical screening has been related to increased rates of adherence.^11, 13, 16, 19, 24^ By using a psychological proactive or preventative approach, health care providers can work towards more women adhering to cervical cancer screening.^8^

#### Barriers and facilitators to cervical screening in the South African context

Historically, there had been a severe shortage of research focussing on individuals of African heritage in South Africa. This is most evident in the paucity of research involving disadvantaged women. Currently, only two studies have investigated cervical cancer screening amongst South African women. The first was limited to the assessment of the degree of knowledge about cervical cancer and screening possessed by disadvantaged women.^20^ The second study assessed the level of knowledge about cervical cancer and screening possessed by South African women from a variety of socio-economic backgrounds, and also assessed the utilisation of screening facilities within these different groups.^26^ There is therefore a need to determine what barriers and facilitators exist which are specific to the currently disadvantaged women living in underserved regions of South Africa. This is particularly important considering the socio-political disadvantages faced by these women.

#### Objectives

The aim of this study was to explore disadvantaged women's perceptions and experiences of cervical screening and cervical cancer. The information generated may contribute to current understanding of the reasons why women do not adhere to recommended screening practices. It is hoped that this knowledge will aid healthcare policy formation and service development by highlighting the reasons for low rates of cervical screening amongst these women.

## Research methods and design

### Theoretical framework

The Health Belief Model (HBM) provided a theoretical framework for this research. This model has consistently shown to be effective when used as a theoretical basis for exploring women's cervical screening practices.^11, 13, 17, 19, 28, 29^

The research questions asked how women's perceptions and knowledge of and their perceptions and experiences regarding barriers and facilitators to cervical screening influenced their decisions to adhere to recommended screening practices. The data generated by this research therefore represent the personal and subjective experiences of these women. The HBM's focus on perceptions, self-efficacy and cues to action/experiences provided a useful framework within which to structure and analyse the present research. In addition, the exploratory nature of this research and the extremely under-researched population it focussed on further supported the decision to use a model that emphasises the importance of perceptions and experiences.

### Setting

The study took place in two informal settlements, Masiphumelele and Red Hill, both within the greater Cape Town area. Though Masiphumelele was originally established as a formal settlement with an accessible healthcare clinic, the influx of migrating workers and the resultant lack of adequate housing have led to poor living conditions and overcrowding. Most of the settlement's inhabitants reside in informal structures. As a consequence of the overcrowding and the expansion of unsafe informal shelters, life in this settlement is characterised by poor service delivery as well as unsanitary living conditions.

Red Hill is a small informal settlement with approximately 1000 residents and only informal housing. The settlement is more integrated than Masiphumelele, with a mix of Black and Coloured residents. In addition, because of the recent influx of foreign migrants into South Africa many of the Black residents in Red Hill are not South African. Red Hill is still characterised by poverty, unemployment, a high risk of fire and unsanitary living conditions. Red Hill does not have any schools and there is only a part-time medical clinic; service delivery in the area is poor.

### Design

Focus groups were used for data collection. Focus groups are a widely utilised method in qualitative research and have found particular popularity within health psychology.^30, 31^ The power of focus groups is found in the way in which they encourage participants to interact, and therefore respond, to one another's opinions. This creates an environment where statements can be challenged and elaborated upon in a manner which provides far richer data.^30, 31^

The nature of focus groups creates an opportunity for the researcher to explore questions about the way in which attitudes are formed or altered. Focus groups also allow for the simultaneous collection of a diverse number of opinions.^32^ In addition, focus groups give the researcher the opportunity to investigate the ways in which the participants co-construct the meanings attached to a specific phenomenon.^31^ For this reason, focus groups are suitable for research which is concerned with eliciting the participants’ own understandings, perspectives and opinions of a phenomenon.^30^

### Procedure

This research involved a total of four focus groups. Two of the focus groups were conducted with women who had undergone cervical screening (one group from Masiphumelele and one from Red Hill) and two were conducted with women who had never undergone cervical screening (again, one group from Masiphumelele and one from Red Hill). The researchers decided to divide the women according to their screening status so as to reduce non-adhering women's feelings of embarrassment or intimidation by the presence of their adhering counterparts. Each participant was contacted by a member of their community who was working with the researchers. The recruiters briefly outlined the research and obtained permission from each participant for their information to be given to the researchers.

The focus groups were held in the recruiters’ homes in the informal settlements of Red Hill and Masiphumelele. These locations were private and provided a familiar setting and therefore served to reduce participant anxiety and encourage open discussion. The focus groups were led by lead researcher through a series of questions for discussion in English. A co-researcher took notes on non-verbal communication. The focus group closed with a debriefing by the lead researcher, and the participants were provided with a list of local cervical screening services. Once the focus groups had been conducted, the researchers transcribed the recordings. As the researchers were interested in the participants’ experiences and perceptions regarding cervical screening, the manner in which the participants expressed themselves when referring to these experiences was of interest.

### Analysis

The data generated by this research was analysed using thematic analysis, a theoretically flexible way in which data, and therefore themes, can be identified, organised, analysed, and reported. Thematic analysis contains six phases.^33^ Two researchers analysed the data in order to reduce the bias occurring when there is a single researcher.

The first phase involved the researchers familiarising themselves with all the transcribed data. This was performed by transcribing, reading and re-reading data. During this phase the researchers jotted down any ideas for coding which were relevant to the research question.

The second phase consisted of the initial construction of codes from the data. All interesting features of the data were coded in a systematic fashion, and relevant data was collated for each code.

During the third phase, the different codes were grouped into potential themes, and the relevant data extracts for each code were collated within the different themes.

The themes were reviewed in the fourth phase. This phase began with the researchers checking to see whether the collated codes for each theme formed a coherent pattern. At this stage, the two researchers consulted with each other on the themes and codes that had emerged. Codes were then organised into a smaller number of agreed-upon main themes, and definitions for each theme were developed. Data saturation was reached when the researchers could no longer identify new information.

In phase five a third and fourth researcher independently reviewed and organised extracts of the data according to the themes and codes which had so far emerged. This established code and theme validity, and the codes and themes were finally defined and named.

The final phase involved producing a concise and coherent description of the research findings for the identified codes and themes. Non-verbal data recognised as being significant to the analysis were considered throughout the process of data analysis.

## Ethical considerations

This research was approved by Department of Psychology's Ethics Committee at the University of Cape Town, and the researchers adhered to the University of Cape Town's guidelines for research with human subjects. Additionally, the research met the ethical requirements specified by the Research Ethics Department of the Department of Psychology.

### Risks and benefits for participants

This research did not pose any great risk to the participants. However, there was a chance that the participants might be distressed if the discussion led them to conclude that their health was at serious risk because of their lack of screening adherence. In order to account for this, researchers provided each individual with the contact information of a counsellor working from Victoria Hospital. This is a government hospital and therefore counselling services are free. Consequently, cost would not be a barrier to seeking further support. This research did not provide any direct benefits to the participants. However, the women were provided with resources on cervical cancer, which encouraged them to undergo screening. In addition, an information session was held within each of the communities. This meeting was attended by a cytopathologist from Groote Schuur hospital who discussed cervical cancer and the procedure for cervical screening. Thereafter the women were given the opportunity to ask questions regarding cervical cancer, screening and treatment.

### Recruitment procedure

Purposive, convenient sampling was used to recruit the participants. Members of two informal settlements located in the southern part of Cape Town, Red Hill and Masiphumelele, assisted the researchers in recruiting participants from their respective communities. A total of 21 Black women between the ages of 21 and 53 years and residing in these informal settlements participated in this study. Only women above 21 years of age were included in this study. This is in accordance with the cervical cancer screening guidelines set by the American National Cancer Institute^27^ which recommend that women begin cervical screening within three years of their first experience of sexual intercourse, or at the age of 21, whichever comes first. This institution's guidelines were used as the South African Department of Health did not have recommended age guidelines available. A basic level of English proficiency was also necessary for inclusion in the study, as the researchers were English-speaking and as a result could not facilitate the research in other languages. A further inclusion criterion was that the participating women had to reside in either Red Hill or Masiphumelele. This was because these were the communities in which the researchers had established contacts and had found private, easily accessible venues.

### Informed consent

Before each focus group discussion commenced, researchers introduced themselves and explained the purpose of the focus group to the participants, as well as the way in which it would be conducted. The researcher leading the focus group then went through the information sheet, answering any questions that the participants had, before asking them to sign the consent form. As the focus groups were tape recorded, the researcher also explained why the tape recorder was being used and how it worked.

### Data protection

The tape recordings were only handled by the researchers involved. These researchers had also signed the consent forms agreeing to ensure that the participants remain anonymous. The transcribing and data analysis were performed by the researchers involved in the project.

## Discussion of results

The thematic analysis began with one of the researchers and a bilingual research assistant transcribing the focus group discussions. The analysed data were reviewed independently by a second and a third researcher, and after a discussion to clarify themes and ensure agreement, the transcripts were coded according to the agreed-upon themes and sub-themes. Three main themes – *barriers, facilitators* and *knowledge* – were identified.

### Barriers to Screening Service

A number of themes relating to barriers to screening service emerged in the analysis of the data. *Barriers* were defined as the negative outcomes and impediments to undertaking health behaviours. Two sub-themes were identified, namely *structural* barriers and *psychosocial* barriers.

*Structural* barriers were factors which affected the accessibility of healthcare services to the women. The *structural* barriers that appeared in this research were those of *time*, *age*, and *health education*.

A lack of *time* because of long working hours appeared to prevent women from attempting to attend screening:

‘I have never get the chance to get to the clinic because I'm always working.’ (Participant 4)

Disadvantaged women living in informal settlements are subjected to the gross inequalities of contemporary South African society. Their experiences of economic pressures and consequently of long working hours are aspects of these.

*Age* emerged as another structural barrier which inhibited women from adhering to a cervical screening programme. Participants often expressed their willingness to go for a Pap-smear, but experienced *age* as being a significant barrier to attendance:

‘OK, why did you decide not to go for a Pap-smear? When you heard about it at the clinic, why didn't you …’ (Researcher)

‘I did, but they said I am underage. They said you must have a 30.’ (Participant 1)

Women who had the time and did attempt to go for a Pap-smear were frequently denied free screening as their age did not make them eligible for this free service. This was because of a government policy of which the researchers were unaware prior to commencing this research.^34^ As a result of limited healthcare resources in South Africa, only women 30 years and older are eligible for free cervical screening. However, women younger than 30 years are eligible for free screening if they are HIV positive. Women living with HIV have an increased risk of developing cervical cancer and an exception is made for them.^35, 36^ Once again, the disadvantaged position of these women within a society marked by inequality is highlighted by their limited access to cervical screening services.

A lack of availability of information about cervical cancer and preventative screening emerged as a key barrier preventing women from adhering to cervical screening:

‘I think that it's information, that info we don't know, and myself I don't realise that it's starting probably, that information doesn't really go to especially, we most of the women, we not really well educated or educated can you say, so there's no one who goes and give us that information that you have to go.’ (Participant 16)

The barrier of a dearth of *health education* again pointed to the disadvantaged position of these women. Many of these women have never, and may never, be given the opportunity to be educated on their health and on illness prevention options. The women identified not being exposed to information regarding cervical cancer and screening as a reason for not attending Pap-smears.

*Psychosocial* barriers were factors related to influential social and psychological elements. The main sub-themes of *psychosocial* barriers were identified as *fear* and *stigma*. Women reported that *fear* relating to undergoing such an invasive procedure deterred them from attending a Pap-smear. Associated with *fear* were feelings of uncertainty and confusion. Women were unsure about why they should undergo the test:

‘I don't think they really explain to people what it is… (silence) so you just do it because the nurse told you to do it, you don't really understand what it is.’ (Participant 9)

Women also articulated their fear of feeling uncomfortable and not knowing enough about the procedure:

‘I am feeling, uhm uh, uncomfortable.’ (Participant 1)

‘We not sure because don't know anything.’ (Participant 12)

In addition, the women were worried about possible *stigma* associated with attending screening. Women mentioned that other community members may speak about them in a negative manner if they were seen going for a Pap-smear:

‘Yes, people's going to say about you when you go there …’ (Participant 1)

‘(slight laughter) I feel fine ‘cos I don't care what they say.’ (Participant 4)

‘It's a secret between you and that person that is actually doing the Pap-smear.’ (Participant 6)

This concern may have been related to negative associations with cervical screening. For instance, the fact that only HIV positive women younger than 30 years are eligible for free cervical screenings, may deter younger women from undergoing screening for fear of revealing their positive statuses.^37, 38^

The emergence of f*ear* as a theme was consistent with previous research which identified fear surrounding the process of Pap-smear testing as contributing to a lack of screening adherence.^11, 13, 14, 16, 17, 18, 19, 24, 25^ Factors such as *health education* and *time*, which were identified across all focus groups, were also cited by other studies as barriers to cervical screening adherence^11, 13, 14, 15, 16, 17^ A significant contributor to the development and perpetuation of these barriers was lack of, or poor, health education. Inaccurate knowledge regarding cervical screening and cervical cancer prevented the women from being able to access their risk accurately. It also contributed to their screening-related fears.

The above *structural* and *psychosocial* factors are in agreement with the factor of *perceived barriers* identified in the HBM as predicting the adoption of health behaviours.^39^ In addition to the above *structural* and *psychosocial* barriers, several facilitators of screening adherence were identified.

#### Facilitators to screening service

Facilitators can be defined as environmental or bodily events which encourage women to attend cervical screening. This theme can be divided into two sub-themes, namely *information sources* and *physical state*.

*Information sources*. Women received their information about cancer and Pap-smears from informal as well as formal sources. *Formal sources* of information included radio and television broadcasts and clinics. These *formal sources* focused on providing information which was specific to cervical cancer and cervical screening and created a desire in the women to either attend screening or to seek more information on cervical cancer and screening. Information received from a clinic was the most frequently cited of these sources.

‘They always talking about the cancer at the clinic, and about the Pap-smear … if you go to the clinic they will tell you about, and there is the signs there they say talking about the Pap-smear…’ (Participant 8)

Radio and television were also often cited as sources of information regarding cervical screening:

‘I go to the clinic because I hear to the radio cervical cancer and the breast cancer; all the women they must wake up and go to the clinic in order to check.’ (Participant 5)

‘I don't understand cervical cancer … I want to know about it, ja, I hearing it on the radio, on or TV, at the clinic, whatever, but I don't know about it, I want to know about it.’ (Participant 9)

‘I've never heard about it ever, the only time I heard about it was on the radio, and that's the only time.’ (Participant 11)

*Informal sources* included relatives who had cancer as well as individuals suffering from cancer in their work environments. Many women spoke about their experiences of witnessing an employer who was diagnosed and treated for cancer. It is noteworthy that when relating their experiences and perceptions gained from *informal sources* the women in the focus groups rarely spoke about cervical cancer; they usually spoke about cancer in general. *Informal sources* increased the women's general awareness of cancer and its potential outcomes:

‘I heard the cancer in 1994 when my mother she start getting sick, and my father he taking to the doctor in East London and they didn't do the Pap-smear, at that time, it was 1994, and she passed away 1995 …’ 9 (Participant 8)

‘My cousin's sister had the breast cancer, and she is still alive, they cut my sister's breast and she's still got a hole on her breast … My boss, she had made the, the operation on May for the breast, is taken off the breast, yes is still alive.’ (Participant 5)

The above findings, illustrating the importance of small media as sources of information for cervical cancer and preventative screening, are consistent with previous research. It has repeatedly been shown that small media are primary sources of formal information. In addition, effective interventions to increase screening adherence have been shown to use small media to some degree.^15, 40, 41, 42^ Furthermore, the source of their information has been revealed to have a significant effect on women's adherence to cervical screening. Most notably, a health provider's recommendation has frequently been found to be a key motivator in adherence, especially within underserved populations.^6, 16, 18, 25^ This is particularly significant when considering the research participants’ current context. Within many informal settlements in South Africa medical service provision is poor and women often do not have adequate access to medical clinics. In light of this, it is of great concern that the clinics were regularly cited as sources of formal information, as a large majority of the women in the informal settlements may not have the opportunity to receive information from clinics.

#### Physical state

The women referred to four main facilitators of cervical screening regarding their *physical state*: the presence of *physical symptoms*, *symptom relief*, the presence of another *physical illness* and the desire to know their *health status*. Women spoke of physical symptoms as encouraging screening adherence, for example:

‘When you are feeling different maybe you got the pains, the abdominal pains, and you go to the doctor and check, to make a check-up…’ (Participant 5)

A commonly cited facilitator of cervical screening was the relief of undesirable physical symptoms:

‘I often got stomach pains as well, but it all stopped after Pap-smears.’ (Participant 7)

‘Because I always had this discharge but then I did the Pap-smear and then it stopped…’ (Participant 14)

‘You check if you have the pains and after you don't have it.’ (Participant 8)

With regard to the presence of another physical illness, this woman spoke of her HIV diagnosis as a facilitator to screening:

‘uhm … I'm going to tell you the truth … I'm going to the clinic … in 2002, because they was talking about the HIV. And I went to the clinic in Sunvalley, it was 2002 in October. I went to test for HIV … they find out that I am positive, that is fine. Now I went to test for Pap-smear.’ (Participant 8)

Finally, many women expressed a desire to know their physical health status and reported that this acted as a facilitator to undergoing screening:

‘Me, to check my womb, to see if it's still perfect in the right place, position, no infection …’ (Participant 15)

‘So for me it works because when I do get the results back I get to see whether I have cancer or anything wrong with my womb.’ (Participant 14)

‘I want to know I'm safe.’ (Participant 2)

The findings that the presence of *physical symptoms* and a desire to know one's *health status* are facilitators to cervical screening adherence are consistent with previous research.^11, 13, 15, 19^ The tendency for women to only seek cervical screening once they are experiencing symptoms has been well documented. Research has shown that women frequently believe Pap-smears to be a test for existing cancer.^14, 15, 18^ As a result women will frequently only undergo screening once they are suffering from a reproductive health problem such as vaginal bleeding or discharge.^11, 15^ This finding further emphasises the importance of information provision as a lack of awareness as to the preventative nature of cervical screening is at the root of this tendency to seek treatment only when physical symptoms are experienced.

The findings that the experience of *symptom relief* subsequent to having a Pap-smear and the presence of another *physical illness* were important facilitators to cervical screening adherence were unique to this research. The relief of undesirable physical symptoms has not been reported as a facilitator to cervical screening in previous research. This finding is of particular interest because Pap-smears are for screening purposes only and are not used for treatment and symptom relief. A possible explanation for the symptom relief experienced by these women is that health providers might have identified and treated infections without adequately informing the patients.

All other references to the presence of an illness as a facilitator to screening are related to a positive HIV status. Taking South Africa's current battle with the HIV epidemic into account, it is not surprising that this has become a facilitator to cervical screening adherence. HIV is associated with an increased risk for the development of cervical cancer, a more advanced and aggressive disease presentation and a poorer prognosis.^35, 36^ For this reason, clinics have prioritised this high-risk population when it comes to cervical screening.

#### Knowledge

An overreaching theme which emerged in the data across all four focus groups was poor *knowledge* about cervical cancer and screening behaviours. If women have inadequate knowledge about prevention and cervical cancer they are not likely to present for screening.^15^ Three sub-themes emerged from the data: *knowledge about disease risk*, *view of cervical cancer*, and *knowledge about Pap-smears*.

*Knowledge about disease risk* was defined as knowledge regarding behaviours believed to place women at risk of developing cervical cancer. Women identified being HIV positive and having sexual intercourse with multiple partners along with smoking, heavy drinking, unhealthy eating and being above the age of thirty as factors placing them at risk for the development of this disease:

‘But I heard that when you are HIV positive, but I heard at the clinic when you are HIV positive you have to check your Pap-smear after you have delivered the baby …’ (Participant 2)

‘I think cervical cancer sometimes is the transmission of the disease, is going to sleep with another … a lady is roaming about, the ladies is sometimes has got the infection, not to go the clinic I thinks can make the cancer.’ (Participant 5)

‘Like smoking …’ (Participant 6)

‘Drinking too much … or eat, you not eat like healthy food …’ (Participant 5)

‘It's common in … mostly in women above the age of 30.’ (Participant 9)

The knowledge that the women conveyed tended to relate to their conceptions of healthy behaviour in a general sense. There was an absence of an accurate and detailed understanding of cervical cancer. This theme has support from the HBM and is directly related to the HBM's factor of *perceived susceptibility*. *Perceived susceptibility* is defined as an individual's assessment of the probability of suffering from a condition which would negatively affect one's health.^39^ In relation to the current research, it is only logical that a woman's knowledge regarding risk factors for cervical cancer would influence her perception of her own personal risk of developing this disease.

*View of cervical cancer*. This described the women's subjective perceptions and feelings about cervical cancer and the seriousness of not being treated. Women felt that death was an inevitable outcome of cancer as they believed that there is no cure for cancer. Additionally, when asked about cervical cancer, women associated vaginal discharge and sexually transmitted diseases (STIs) with the illness:

‘If they do find it in any part of the body, there's no cure for it, you die when they have found the cure too late.’ (Participant 6)

‘She died and it was too late to get a cure …’ (Participant 7)

‘Just cancer, do you think about anything else with the Pap-smear?’ (Researcher)

‘Discharge …’ (Participant 3)

‘STIs …’ (Participant 1)

This theme, *view of cancer*, can be seen to influence behaviour decisions in a similar manner to the factor of *perceived severity* in the HBM.

#### Knowledge about Pap-smears

Some of the women had accurate knowledge of the Pap-smear test procedure, but the majority failed to identify the test's function correctly. The women believed that Pap-smears were a means of reducing bodily pains and discharge, as well as being necessary prior to sterilisation. A number of women had experienced pain or discharge which had disappeared after they had had a Pap-smear:

‘I've stopped having hip pains after doing Pap-smears.’ (Participant 6)

‘I often got stomach pains as well, but it all stopped after Pap-smears.’ (Participant 7)

‘You check if you have the pains and after you don't have it …’ (Participant 8)

‘Because I always had this discharge but then I did the Pap-smear and then it stopped …’ (Participant 14)

Many of the women believed that a Pap-smear test was a positive test which would enhance their health status:

‘Pap-smear makes everything right.’ (Participant 6)

‘Me, to check my womb, to see if it's still perfect in the right place, position, no infection …’ (Participant 15)

‘Your result is abnormal, you find out you've got it, you've got the cervical cancer, if the result is normal you've got the normal, you are healthy …’ (Participant 5)

Most of the participants had inaccurate knowledge regarding the function and benefits of a Pap-smear test. This finding is consistent with previous research which has identified a lack of awareness of screening benefits as a barrier to screening adherence.^14^ Moreover, these findings support the already existing research which highlights a lack of knowledge as a key barrier to cervical screening in South Africa.^20^ This further emphasises the importance of increased awareness and knowledge of cervical screening as a means of improving adherence amongst women living in South Africa's informal settlements.

#### Reflexivity

Our sociocultural position as white, English-speaking, young middle-class females, with a starkly different culture from that of our participants, may have affected the type of information the participants felt comfortable sharing with us. As the focus groups progressed it became apparent that the participants assumed we were associated with a hospital or medical system as they often asked for medical explanations and information. Consequently, we remained separate and became intensely aware of our status as ‘outsiders’. This was exacerbated by our inability to communicate effectively with the women in their own language, isiXhosa. In addition, there was a mutual understanding between the women and a manner of relating amongst themselves from which we were excluded.

As our cultural background prioritises and places importance on westernised medical care, it was important for us to be mindful of this whilst working with the data. Constant reflection on our perceptions of ‘normal’ health adherence behaviour was necessary, in order to conduct and interpret the research and data without imposing our personal views on the meanings that emerged. Although our aim was not to control or interfere with the research process, we acknowledge that our social and cultural positions could have impacted on research outcomes.

## Limitations of the study

As language constructs and shapes rather than describes an individual's reality and experiences, a limitation of this research was its reliance on language.^31^ In the proposed research the researchers’ stipulation that the focus groups be conducted in English inhibited the extent to which the women could comprehensively relate their experiences. Many of the participants’ English proficiency was limited, and for this reason the experiences conveyed to the researchers were constrained, as were the researchers’ ability to respond and reflect appropriately when the women expressed themselves in isiXhosa. This being said, the researchers were aware that the accounts they received were only interpretations and representations of what the women actually experienced.

The participant sample was small and only representative of women living in disadvantaged communities in the greater Cape Town area. These limitations restrict the transferability of the research results to women living in similar communities in other parts of South Africa. However, the information gleaned is important for informing healthcare providers and future researchers about some of the potential issues that require consideration and further exploration.

### Recommendations

One recommendation for future research would be to create a greater community buy-in. There is a need to establish connections with various important community members and stakeholders before research commences. This will allow for the community to become comfortable with the researchers’ presence whilst at the same time giving the researchers credibility. Secondly, the use of co-researchers who are members of the communities and who would be trained as focus group facilitators would be an ideal solution to the problems of language and the researcher's ‘outsider’ status. If this is not possible, the focus groups’ facilitators must be proficient in the African language predominantly spoken by the group's participants.

## Conclusion

This research revealed several interesting findings about the experiences of disadvantaged South African women in relation to cervical cancer and preventative screening. Three broad factors, namely *barriers*, *facilitators* and *knowledge*, were found to influence a woman's decision to adhere to screening tests.

The barriers identified were divided into two groups, namely *structural* and *psychosocial* barriers. *Structural* barriers included *time*, *age*, and *health education*. *Psychosocial* barriers included *fear* and *stigma*. A crucial structural barrier, specific to the South African context, is the fact that only women who are HIV positive or older than 30 years are eligible for free cervical screening. Related to this structural barrier was the *psychosocial* barrier of *fear*. Two dominant fears associated with clinic attendance emerged. The first was a fear of undergoing an invasive examination and the second was a fear of being stigmatised by one's community. As previously mentioned, women under the age of 30 years only receive free Pap-smear testing if they are HIV positive and therefore being screened can amount to disclosing one's status.

Another influential theme which emerged across all four focus groups was *knowledge*. A particularly worrying aspect of this factor was a widespread lack of awareness as to the purpose of the Pap-smear test. An absence of *health education* emerged as a key reason for this lack of awareness. What knowledge the women did have was predominantly gleaned through *formal sources* such as clinics and small media. These formal forms of information delivery emerged as being most effective. Interventions aimed at improving adherence amongst these populations should focus on utilising these formal sources of information in their health promotion communications. The risks, benefits and purpose of screening need to be thoroughly explained to all the women who attend clinics.

In addition to information sources, the women related that the presence of *physical symptoms*, *symptom relief*, the presence of another *physical illness*, and the desire to know ones physical *health status*, were all facilitators of screening adherence. The first two sub-themes further emphasised that a lack of knowledge is a key barrier to screening within this population as underlying both of these facilitators is inaccurate information regarding the purpose of a Pap-smear. This absence of knowledge needs to be addressed if screening adherence is to be improved. Furthermore, the benefits of screening, such as knowing one's physical health status, as well as the risk factors particular to cervical cancer, will need to be highlighted.

To summarise: although this study has highlighted many obstacles to cervical screening, it has also identified several factors which are crucial for improving adherence. Stigma reduction and culturally specific health education promise to be effective ways of increasing the cervical screening adherence of disadvantaged women residing in urban informal settlements. Changes in health policy to lower the age at which free screening is available are also essential to increasing screening adherence amongst this underserved population. Allowing younger women easy access to preventative screening will improve prognoses, treatment will become more cost-effective, and the unnecessary suffering experienced by too many will be substantially reduced.
